# The role of ACE1 I/D and ACE2 polymorphism in the outcome of Iranian COVID-19 patients: A case-control study

**DOI:** 10.3389/fgene.2022.955965

**Published:** 2022-09-05

**Authors:** Arezoo Faridzadeh, Mahmoud Mahmoudi, Sara Ghaffarpour, Mohammad Saber Zamani, Akram Hoseinzadeh, Mohammad Mehdi Naghizadeh, Tooba Ghazanfari

**Affiliations:** ^1^ Immunology Research Center, Mashhad University of Medical Sciences, Mashhad, Iran; ^2^ Department of Immunology and Allergy, School of Medicine, Mashhad University of Medical Sciences, Mashhad, Iran; ^3^ Immunoregulation Research Center, Shahed University, Tehran, Iran; ^4^ Department of Immunology, Faculty of Medicine, Mashhad University of Medical Sciences, Mashhad, Iran; ^5^ Noncommunicable Diseases Research Center, Fasa University of Medical Science, Fasa, Iran; ^6^ Department of Immunology, Shahed University, Tehran, Iran

**Keywords:** polymorphism, insertion/deletion, angiotensin-converting enzyme (ACE), coronavirus disease 2019 (COVID-19), severity, SNP

## Abstract

**Background:** Since the beginning of the pandemic of coronavirus disease 2019 (COVID-19), many countries have experienced a considerable number of COVID-19 cases and deaths. The etiology of a broad spectrum of symptoms is still debated. Host genetic variants might also significantly influence the outcome of the disease. This study aimed to evaluate the association of angiotensin-converting enzyme (ACE1) gene Insertion/Deletion (I/D) polymorphism (rs1799752) and ACE2 gene rs1978124 single nucleotide polymorphism with the COVID-19 severity.

**Methods:** This study was conducted on 470 COVID-19 patients and a control group of 56 healthy individuals across several major cities in Iran. The blood sample and clinical data were collected from the participants, and their ACE1 I/D and ACE2 rs1978124 polymorphisms were determined using polymerase chain reaction and PCR-RFLP, respectively. Serum levels of C-reactive protein (CRP), interleukin 6 (IL-6), and ACE1 were measured in the blood samples.

**Results:** We found that the ACE1 DD genotype frequency was inversely correlated with the risk of intubation (*p* = 0.017) and mortality in COVID-19 patients (*p* = 0.049). Even after adjustment, logistic regression demonstrated that this significant inverse association remained constant for the above variables at odds ratios of (OR) = 0.35 and Odds Ratio = 0.49, respectively. Also, in the expired (*p* = 0.042) and intubated (*p* = 0.048) groups with II + ID genotypes, the mean level of CRP was significantly higher than in the DD genotype group. Furthermore, in both intubated and expired groups, the mean serum level of ACE1 was higher compared with non-intubated and survived groups with II or II + ID genotypes. The results also indicated that ACE2 rs1978124 TT + CT genotypes in females have a significant positive role in susceptibility to COVID-19; however, in females, the TT + CT genotypes had a protective effect (OR = 0.098) against the severity of COVID-19.

**Conclusion:** These findings suggest that ACE1 I/D and ACE2 rs1978124 polymorphism could potentially influence the outcome of COVID-19 in the Iranian population.

## 1 Introduction

The current pandemic results from severe acute respiratory syndrome coronavirus 2 (SARS-CoV-2), causing coronavirus disease 2019 (COVID-19). It has turned into a full-blown global crisis since its onset, prompting researchers worldwide to seek solutions for this problem. So far, COVID-19 has affected over 566 million individuals worldwide, resulting in 6.3 million deaths (WHO, 2022). The clinical spectrum of SARS-CoV-2 patients ranges from asymptomatic and mild flu-like symptoms to severe acute respiratory distress syndrome (ARDS). The prevalence and mortality rates of COVID-19 vary considerably from country to country, which raises the question of whether geographical origin and host genetic variations play a role in the severity and mortality of COVID-19 infection.

Angiotensin-converting enzyme-2 (ACE2) is the receptor of SARS-CoV-2, and transmembrane protease serine 2 (TMPRSS2) facilitates the virus entry. ACE2 is mainly expressed in the lung, intestine, cardiovascular system, kidney, adipose tissue, and central nervous system (Gheblawi et al., 2020). ACE1 converts Angiotensin I into Angiotensin II, which promotes inflammation, thrombosis, and vasoconstriction. ACE2 converts Angiotensin II into Angiotensin 1–7 and hence promotes vasodilation (D'Ardes et al., 2020). Downregulation of ACE2 expression due to SARS-CoV-2 infection may prevent viral infection. However, it also diminishes the beneficial impacts of ACE2 in the lungs and other organs (Gao et al., 2022). Therefore, COVID-19 may lead to ACE1/ACE2 imbalance and increase angiotensin II levels because it activates the renin-angiotensin-aldosterone system (RAAS) and thus the progression of COVID-19, especially in patients with underlying diseases such as high blood pressure (HTN), cardiovascular disease (CVD) and diabetes (DM) (Adamzik et al., 2007; Beyerstedt et al., 2021a). Moreover, factors like sex, age, smoking habit, obesity, blood group, HTN, DM, CVD, and genetics might be important in COVID-19 infection (Gard, 2010; Cai et al., 2020; Ejaz et al., 2020; Ovsyannikova et al., 2020; Sattar et al., 2020; Zeberg and Pääbo, 2020; Goel et al., 2021). On the other hand, genetic variation of a gene likely modifies the function and expression of an encoded product, which could be considered the interindividual differences in susceptibility to several infectious diseases. So far, few studies have shown the roles of angiotensin-converting enzyme 1 (ACE1), ACE2, and transmembrane protease serine 2 (TMPRSS2) gene variants in the COVID-19 severity (Gorbalenya et al., 2020; Hou et al., 2020; de Araújo et al., 2022; Gintoni et al., 2022).

### 1.1 ACE1

The ACE1 gene is located on chromosome 17q35 with 26 exons and 25 introns. The insertion/deletion (I/D) ACE1 polymorphism (rs1799752) is described by an insertion (allele I) or deletion (allele D) of a 287-base pair (bp) Alu repeat sequence in the 16th intron of the ACE1 gene, which accounts for most of the interindividual variability in circulating ACE activity and shows significant geographic variability. Therefore, I/D polymorphism has three different genotypes: II, ID, and DD (Rigat et al., 1990; Rieder et al., 1999; Sayed-Tabatabaei et al., 2006). Some recent studies suggest that the ACE1 ID polymorphism as a main geographical variation could be one of the genetic markers of susceptibility and pathogenicity of COVID-19 (Pati et al., 2020; Yamamoto et al., 2020). A review on I/D polymorphism suggested that the DD genotype in COVID-19 patients might cause severe lung injury (Zheng and Cao, 2020). However, a meta-analysis by Delanghe et al. demonstrated a negative association between COVID-19 mortality and D alleles frequency from an evaluation of 25 countries in the Middle East, North Africa, and Europe (Delanghe et al., 2020a). An ecological study demonstrated the distribution of II genotype is highest in Asian countries and lower among the African and European countries across 25 countries (Aung et al., 2020). A case-control study in one of the southeastern cities of Iran with a smaller sample size has shown that the II genotype decreases the risk of COVID-19 infection (Kouhpayeh et al., 2021).

### 1.2 ACE2

ACE2 rs1978124 SNP is located on chromosome Xp22 in intron one, suggesting that this variant may affect the expression of the ACE2 gene (Zhao et al., 2010; Patel et al., 2014). Also, some studies demonstrated that the ACE2 rs1978124 SNP was associated with the severity of COVID-19, the risk of diabetes-related left ventricular remodeling, and dyslipidemia (Liu et al., 2018; Sabater Molina et al., 2022).

The present study is the first to determine the potential role of ACE1/ACE2 gene polymorphisms in susceptibility to COVID-19 and the disease outcome of COVID-19 with a larger sample size compared to previous studies, representing the entire Iranian population.

## 2 Participants and methods

### 2.1 Study subjects

This case-control study was conducted on 470 patients with COVID-19 and 56 healthy controls referred to hospitals between 2020 and 2021 across several major cities in Iran. COVID-19 infections were confirmed by real-time reverse transcription-polymerase chain reaction (RT-PCR) or chest CT scan findings. COVID-19 severity was classified into mild, moderate, severe, and critical as defined by the World Health Organization (WHO) (WHO, 2021). The research protocol was approved by the National Institute for Medical Research Development (IR.NIMAD.REC.1399.041).

### 2.2 Data collection and blood sampling

The relevant personal information and medical history of most subjects, including their sex, age, smoking status, and comorbidities, were obtained by a patient checklist. Informed consent was acquired from all individuals or their family members before collecting blood samples. The selection method of patients was not probabilistic. At the beginning of hospitalization, all patients and healthy control individuals donated 5 ml blood samples collected in the clot activator tubes and tubes containing ethylene diamine tetraacetic acid (EDTA).

### 2.3 Interleukin-6, C-reactive protein, and ACE1 assessment

Interleukin-6 (IL-6) was measured in serum samples using an automated immunoassay (IMMULITE 2000; Siemens Healthcare Diagnostics, The United Kingdom). Serum level of C-reactive protein (CRP) and the quantitative enzymatic determination of ACE1 were done in serum samples with 7180 clinical analyzers (Hitachi, Japan) using ACE BIOLIS (Genbio, Ireland).

### 2.4 DNA extraction and genotyping

DNA was extracted from the buffy coat samples of all subjects using a spin column kit (GenAll Exgene Cell SV mini kit, GenAll Biotechnology, South Korea).

#### 2.4.1 Detection of ACE I/D gene polymorphism

**FIGURE 1:**
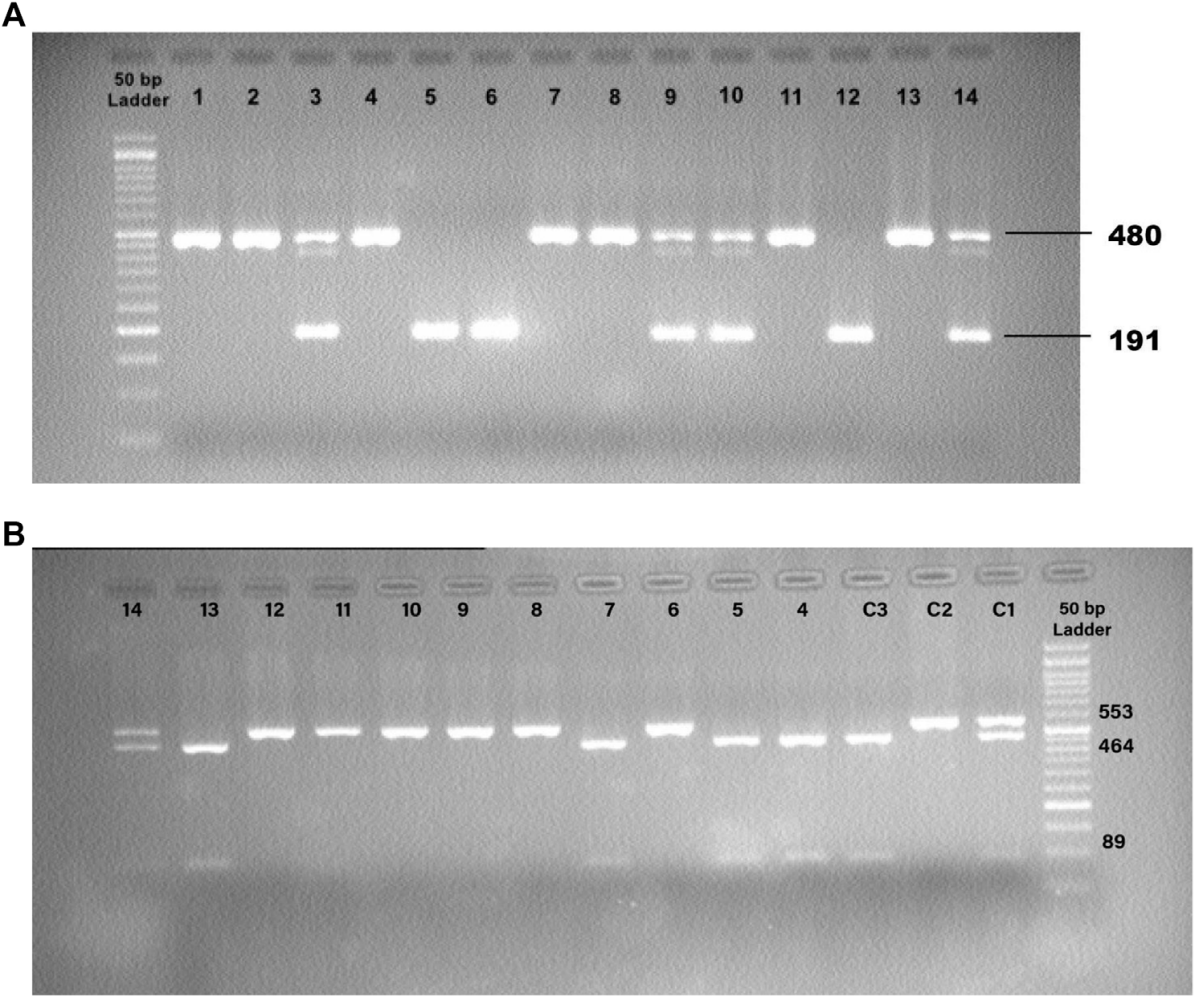
**(A)** Detection of the PCR Products for ACE1 Insertion/Deletion (I/D) Polymorphism. 12: Control, DD; 13: Control, II; 14: Control, ID; 1,2,4,7,8,11: II; 5,6: DD; 3,9,10: ID. **(B)** Detection of the PCR-RFLP Products for ACE2 rs1978124 SNP. C1: Control, CT; C2: Control, TT; C3: Control, CC; 4, 5, 7, 13: CC; 6, 8, 9, 10, 11, 12: TT; 14: CT.

The ACE1 intronic Alu insertion (I) or deletion (D) polymorphism (rs1799752) was determined by polymerase chain reaction (PCR) and agarose gel electrophoresis methods with specific primers (forward primer- 5′ -CTG​GAG​ACC​ACT​CCC​ATC​CTT​TCT-3′ and reverse primer- 5′ -GAT​GTG​GCC​ATC​ACA​TTC​GTC​AGA​T-3′). PCR reactions were performed in a final volume of 25 μL comprising TEMPase Hot Start 2x Master Mix A (Ampliqon, Denmark), ten pmol of each primer (TAG Copenhagen A/S, Denmark), 20–100 ng genomic DNA, and distilled water. After the initial denaturation step at 95°C for 15 min, the reaction mixtures were subjected to 40 cycles of 95°C for 30 s, 58°C for 45 s, 72°C for 1 min, and a final extension at 72°C for 5 min. The PCR products were electrophoresed and visualized in 2% agarose gels containing DNA-safe stains. This technique provided amplification products of 191 bp for the DD genotype, 480 base pairs (bp) for the II genotype, and 480 bp + 191 bp for the ID genotype (Figure 1).

#### 2.4.2 Determination of rs1978124 SNP of the ACE2 gene

**FIGURE 2:**
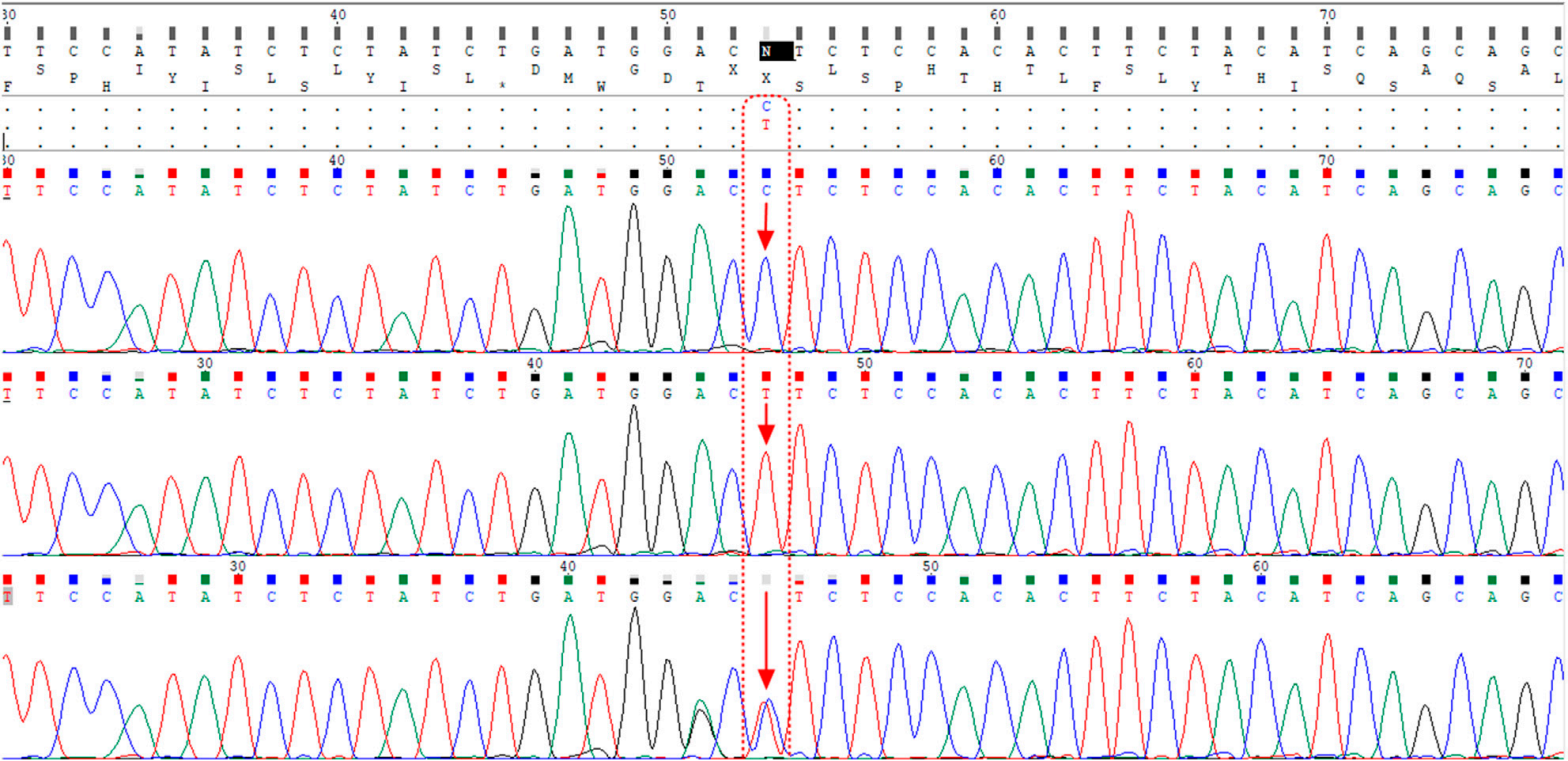
Alignment of the sequencing results of rs1978124 SNP located in ACE2 gene; the first, second, and third rows are related to the sample with CC genotype, TT genotype, and CT genotype, respectively. The presence of both peaks for both alleles is evident in the third row.

The ACE2 rs1978124 polymorphism was assessed by PCR–restriction fragment length polymorphism (PCR-RFLP). The PCR product of 553 bp was generated using the forward primer-5′- CAACCACACATACCACAAT-3′and reverse primer-5′- TTT​CCT​TTA​GCC​TAC​AAT​ATC​AAT -3′, and were incubated with 1 μL of *Echo471* (*Ava II*) restriction enzyme at 37°C overnight. After digestion, two 464 bp and 89 bp products identify the C allele, and a 553 bp band identifies the T allele on agarose gel (Figure 2). About 10% of the samples were directly Sanger sequenced to ensure PCR-RFLP for SNP rs1978124. Using PCR primers, sanger sequencing was performed on 10% of the resulting samples. The sequencing result of rs1978124 SNP after alignment is shown in Supplementary Figure S1.

### 2.5 Statistical analysis

The numerical variables of each group were presented as mean ± standard deviation, and the Mann-Whitney test compared continuous data. The genotypes frequencies were reported as number (percentages) or n (%) in each group and assessed using the Chi-square. Also, Hardy–Weinberg equilibrium (HWE) was calculated and tested by the Chi-square test. The associations of ACE1 insertion/deletion polymorphism and ACE2 rs1978124 SNP with susceptibility and severity for SARS-CoV-2 infection at both the multiple and univariate levels were assessed by multinomial or binary logistic regressions to calculate odds ratios (ORs) (adjusted and unadjusted) with 95% confidence intervals (CI). Individuals were included in the comparison among groups after adjustment for sex, age, HTN, diabetes mellitus, CVD, renal disease, and cigarette smoking. For rs1978124, males and females were analyzed separately since the ACE2 gene is on the X chromosome. A *p*-value less than 0.05 was regarded to be significant.

## 3 Results

### 3.1 Demographic characteristics of the study on Iranian population

Patients were divided into groups based on the disease severity (COVID-19 Treatment Guidelines, 2019). The frequencies of comorbidities such as HTN, diabetes mellitus, CVD, renal disease, and cigarette smoking are presented in Table 1 and Supplementary Table S1. The results of this study demonstrated that HTN (*p* < 0.001), diabetes mellitus (*p* < 0.001), CVD (*p* < 0.001), and renal disease (*p* = 0.024) were significantly higher in COVID-19 patients compared to controls. Also, HTN (*p* = 0.008), diabetes mellitus (*p* = 0.007), and CVD (*p* = 0.009) were significantly correlated with COVID-19 mortality. There was no significant gender difference between the groups. The mean age was associated with increased severity and disease mortality in COVID-19. The mean age of healthy control, outpatients, inpatients, ICU admitted patients, intubated, and expired patients were 45.3 ± 13.3, 44.6 ± 14.1, 57.5 ± 16.5, 61.8 ± 17, 62 ± 14.9, and 65.8 ± 13.9 years, respectively.

**TABLE 1:** Demographic characteristics of the study participants.

	Control (n = 56)	COVID-19				
		Outpatient (n = 207)	Inpatient (n = 263)	Admitted to ICU (n = 76)	Intubated (n = 47)	Expired (n = 57)
Sex (Female ratio)	31 (55.4%)	83 (40.1%)	109 (41.4%)	31 (40.8%)	18 (38.3%)	21 (36.8%)
Cigarette smoking	2 (3.6%)	10 (4.8%)	25 (9.5%)	8 (10.5%)	4 (8.5%)	4 (7%)
HTN	2 (3.6%)	24 (11.6%)	105 (39.9%)	30 (39.5%)	17 (36.2%)	24 (42.1%)
DM	1 (1.8%)	16 (7.7%)	84 (31.9%)	26 (34.2%)	17 (36.2%)	20 (35.1%)
CVD	0 (0%)	5 (2.4%)	70 (26.6%)	20 (26.3%)	9 (19.1%)	14 (24.6%)
Renal disease	0 (0%)	8 (3.9%)	21 (8%)	9 (11.8%)	5 (10.6%)	6 (10.5%)

N: number, %: frequency. Abbreviations: COVID-19, Coronavirus disease 2019; HTN, hypertension; DM, diabetes mellitus; CVD, cardiovascular disease.

### 3.2 ACE1 I/D and ACE2 rs1978124 genotypes

The genotype frequencies of the ACE1 I/D and ACE2 rs1978124 polymorphisms in the patients and control groups agreed with the Hardy-Weinberg equilibrium using the Chi-square analysis. (Supplementary Table S2).

### 3.3 Statistical comparisons between controls and patients (P value) for ACE1 and ACE2 genotypes/alleles

We found no significant relationship between different ACE1 I/D and ACE2 rs1978124 genotypes/alleles frequencies with comorbidities. The relevant statistical details are presented in Supplementary Table S3.

#### 3.3.1 Susceptibility to COVID-19 infection

There was no significant association between ACE1 I/D (rs1799752) and ACE2 rs1978124 SNP genotypes/allele frequencies and susceptibility to COVID-19, but ACE2 rs1978124 T allele and TT+CT genotypes frequencies were higher in women with COVID-19 than in female controls. After adjusting for sex, age, HTN, diabetes mellitus, CVD, renal disease, and cigarette smoking, the comparison of 470 COVID-19 patients and 56 healthy controls through logistic regression revealed no significant association between genotypes of ACE1 and susceptibility to COVID-19 infection. However, for ACE2 CT + TT genotypes, this significant association remained even after adjustment (*p* = 0.008, 95%CI = 5.99) (Table 2).

**TABLE 2:** Association of ACE1 I/D and ACE2 rs1978124 Genotypes/Alleles Distribution with Susceptibility to COVID-19, Adjusted by Age, Sex, Cigarette Smoking, Diabetes Mellitus, HTN, CVD, and renal diseases.

Genotypes alleles N (%)		Study group			Unadjusted		Adjusted	
		Control (n = 56)	COVID-19 (n = 470)	*p*-value (Chi-square)	*p*-value	OR-95%CI- (L-U)	*p*-value	OR-95%CI- (L-U)
**ACE1 I/D**	II	9 (16.1%)	107 (22.8%)	—	—	—	—	—
	ID	30 (53.6%)	215 (45.7%)	0.428	0.204	0.603 (0.276–1.315	0.435	0.722 (0.318–1.636)
	DD	17 (30.4%)	148 (31.5%)	—	0.470	0.732 (0.314–1.705)	0.958	1.024 (0.417–2.515)
	II	9 (16.1%)	107 (22.8%)	—	—	—	—	—
	ID + DD	47 (83.9%)	363 (77.2%)	0.253	0.256	0.650 (0.308–1.368)	0.262	0.823 (0.376–1.802)
	II + ID	39 (69.6%)	322 (68.5%)	—	—	—	—	—
	DD	17 (30.4%)	148 (31.5%)	0.863	0.863	1.054 (0.578–1.925)	0.418	1.306 (0.684–2.491)
	I	48 (42.9%)	429 (45.6%)	—	—	—	—	—
	D	64 (68.8%)	511 (54.4%)	0.576	0.576	0.893 (0.601–1.327)	—	—
**ACE2 rs1978124**	**Female**	—	—	—	—	—	—	—
	CC	6 (19.4%)	7 (3.6%)	—	—	—	—	—
	CT	14 (45.2%)	72 (37.5%)	**<0.001**	**0.018**	4.408 (1.285–15.105)	0.051	3.968 (0.992–15.871)
	TT	11 (35.5%)	113 (58.9%)	—	**<0.001**	8.805 (2.513–30.853)	**0.003**	8.452 (2.087–34.236)
	CC	6 (19.4%)	7 (3.6%)	—	—	—	—	—
	TT + CT	25 (80.6%)	185 (96.4%)	**<0.001**	**0.002**	6.343 (1.973–20.389)	**0.008**	5.994 (1.587–22.25)
	CC + CT	20 (64.5%)	79 (41.1%)	—	—	—	—	—
	TT	11 (35.5%)	113 (58.9%)	**0.015**	**0.018**	2.601 (1.180–5.730)	**0.017**	2.740 (1.197–6.273)
	C	26 (41.9%)	86 (22.4%)	—	—	—	—	—
	T	36 (62.9%)	298 (77.6%)	**<0.001**	**0.001**	2.503 (1.432–4.375)	—	—
	**Male**	—	—	—	—	—	—	—
	C	6 (24%)	73 (26.3%)	—	—	—	—	—
	T	19 (76%)	205 (73.7%)	0.805	0.807	0.887 (0.341–2.307)	—	—

The significant *p* values are in bold, N (%): number (percentage). Abbreviations: ACE, Angiotensin-converting enzyme; I, insertion; D, deletion; OR, odds ratio; CI, confidence interval; L, lower; U, upper; COVID-19, Coronavirus disease 2019.

#### 3.3.2 Severity of COVID-19

Different genotypes/alleles of ACE1 rs1799752 and ACE2 rs1978124 are not associated with COVID-19 hospitalization. After adjustment, binary logistic regression showed that patients with the TT + CT genotypes of rs1978124 carried a lower risk of hospitalization (94.5% vs 98.8%; *p* = 0.042, OR = 0.099; Supplementary Table S4).

**FIGURE 3:**
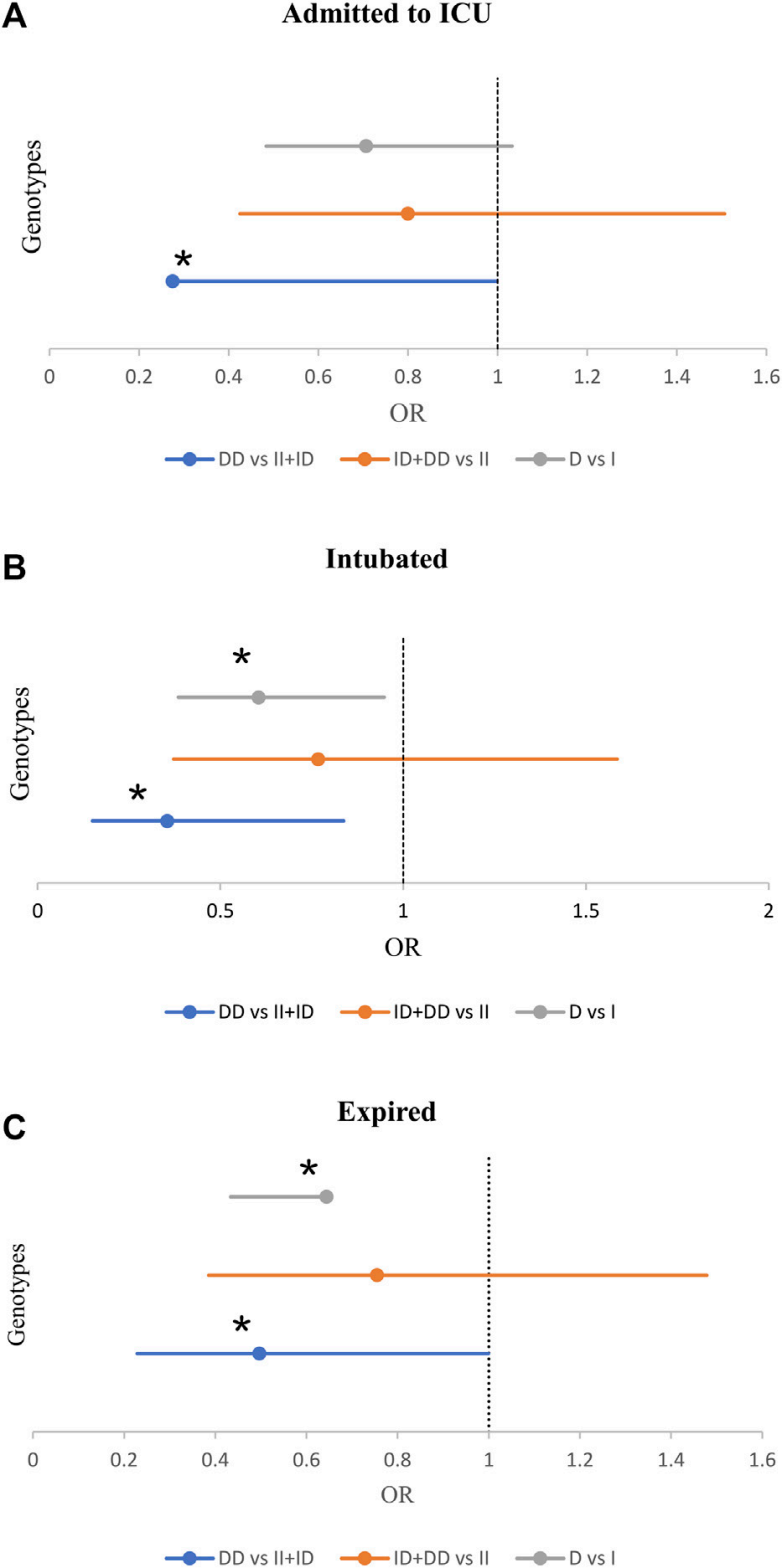
The Forest Plot displays Adjusted Odds Ratio (OR) ± 95% Confidence Intervals (CI) of ACE1 Polymorphism for ICU Admission **(A)**, Intubated **(B)**, and Expired **(C)** Groups; ^*^ *p* < 0.05. (Adjusted by Age, Sex, Cigarette Smoking, Diabetes Mellitus, Hypertension, Cardiovascular Disease, and Renal Diseases).

Also, we observed a significant inverse association between the frequency of DD genotype and the risk for ICU admission (21.1% vs 33.2%, *p* = 0.049, OR = 0.53; Supplementary Table S5). Moreover, there was a significant inverse association between the frequency of DD genotype and intubation (84% vs 66.8, *p* = 0.017). While, the frequency of DD genotype and D allele significantly decreased in intubated patients (14.9% vs 32.9%, *p* = 0.014; 42.6% vs 55.1%, *p* = 0.027). After adjustment, patients with the DD genotype were at a reduced risk of intubation (OR = 0.35, *p* = 0.018; Table 3 and Figure 3).

**TABLE 3:** Association of ACE1 Genotypes Distribution with Intubation of COVID-19 Patients, Adjusted by Age, Sex, Cigarette Smoking, DM, HTN, CVD, and renal diseases.

Genotypes alleles N (%)		Intubation			Unadjusted		Adjusted	
		No (n = 216)	Yes (n = 47)	*p*-value (Chi-square)	*p*-value	OR-95%CI- (L-U)	*p*-value	OR-95%CI- (L-U)
**ACE1 I/D**	II	49 (22.7%)	14 (29.8%)	—	—	—	—	—
	ID	96 (44.4%)	26 (55.3%)	**0.050**	0.887	0.948 (0.454–1.977)	0.808	1.100 (0.510–2.375)
	DD	71 (32.9%)	7 (14.9%)	—	**0.033**	0.345 (0.130–0.917)	0.053	0.376 (0.140–1.015)
	II	49 (22.7%)	14 (29.8%)	—	—	—	—	—
	ID + DD	167 (77.3%)	33 (70.2%)	0.301	0.303	0.692 (0.343–1.395)	0.475	0.767 (0.372–1.585)
	II + ID	145 (67.1%)	40 (85.1%)	—	—	—	—	—
	DD	71 (32.9%)	7 (14.9%)	**0.014**	**0.014**	0.357 (0.152**–**0.838)	**0.018**	0.354 (0.150**–**0.837)
	I	194 (44.9%)	54 (57.4%)	—	—	—	—	—
	D	238 (55.1%)	40 (42.6%)	**0.027**	**0.027**	0.604 (0.385–0.948)	—	—
**ACE2 rs1978124**	**Female**	—	—	—	—	—	—	—
	CC	5 (5.5%)	1 (5.6%)	—	—	—	—	—
	CT	30 (33.0%)	8 (44.4%)	0.637	0.895	1.333 (0.136–13.092)	0.727	1.527 (0.142–16.421)
	TT	56 (61.5%)	9 (50.0%)	—	0.850	0.804 (0.804–7.697)	0.941	0.915 (0.087–9.677)
	CC	5 (5.5%)	1 (5.6%)	—	—	—	—	—
	TT + CT	86 (94.5%)	17 (94.4%)	0.992	0.992	0.988 (0.109–9.002)	0.911	1.141 (0.114–11.449)
	CC + CT	35 (38.5%)	9 (50.0%)	—	—	—	—	—
	TT	56 (61.5%)	9 (50.0%)	0.362	0.365	0.625 (0.226–1.726)	0.391	0.631 (0.221–1.806)
	C	40 (22.0%)	10 (27.8%)	—	—	—	—	—
	T	142 (78.0%)	26 (72.2%)	0.449	0.451	0.732 (0.326–1.645)	—	—
	**Male**	—	—	—	—	—	—	—
	C	35 (28.0%)	6 (20.7%)	—	—	—	—	—
	T	90 (72.0%)	23 (79.3%)	0.422	0.259	1.491 (0.560–3.971)	—	—

The significant *p* values are in bold, N (%): number (percentage). Abbreviations: ACE, Angiotensin-converting enzyme; I, insertion; D, deletion; OR, odds ratio; CI, confidence interval; L, lower; U, upper; HTN, hypertension; DM, diabetes mellitus; CVD, cardiovascular disease.

#### 3.3.3 Mortality of COVID-19

Of the 470 patients, 413 (88.3%) survived, and 57 (12.1%) expired. The D allele and DD genotype frequency were lower in the expired group than in the surviving group (19.3% vs 32.3%, *p* = 0.035; 44.7% vs 55.7%, *p* = 0.028, respectively). After adjustment, patients with DD genotype were at a decreased risk of mortality (OR = 0.49; *p* = 0.049; Table 4 and Figure 3). However, there was no significant relationship between genotype/allele frequency of ACE2 rs1978124 and mortality, even after adjustment (Table 4).

**TABLE 4:** Association of ACE1 Genotypes Distribution with COVID-19 Mortality, Adjusted by Age, Sex, Cigarette Smoking, DM, HTN, CVD, and renal diseases.

Genotypes alleles N (%)		Mortality			Unadjusted		Adjusted	
		Survived (n = 413)	Expired (n = 57)	*p*-value (Chi-square)	*p*-value	OR-95%CI- (L-U)	*p*-value	OR-95%CI- (L-U)
**ACE1 I/D**	II	90 (21.8%)	17 (29.8%)	—	—	—	—	—
	ID	186 (45%)	29 (50.9%)	0.088	0.563	0.825 (0.431–1.580)	0.938	0.972 (0.477–1.982)
	DD	137 (33.2%)	11 (19.3%)		**0.037**	0.425 (0.190–0.950)	0.088	0.470 (0.197–1.120)
	II	90 (21.8%)	17 (29.8%)
	ID + DD	323 (78.2%)	40 (70.2%)	0.175	0.175	0.656 (0.355–1.211)	0.413	0.755 (0.386–1.478)
	II + ID	276 (66.8%)	46 (80.7%)
	DD	137 (33.2%)	11 (19.3%)	**0.035**	**0.035**	0.482 (0.242–0.959)	**0.049**	0.497 (0.229–0.999)
	I	366 (44.3%)	63 (55.3%)
	D	460 (55.7%)	51 (44.7%)	**0.028**	**0.028**	0.644 (0.434–0.644)
**ACE2 rs1978124**	**Female**	—	—	—	—	—	—	—
	CC	6 (3.5%)	1 (4.8%)	—	—	—	—	—
	CT	65 (38%)	7 (33.3%)	0.893	0.704	0.646 (0.068–6.167)	0.443	0.394 (0.036–4.263)
	TT	100 (58.5%)	13 (61.9%)	—	0.824	0.780 (0.087–7.001)	0.593	0.530 (0.052–5.417)
	CC	6 (3.5%)	1 (4.8%)	—	—	—	—	—
	TT + CT	165 (96.5%)	20 (95.2%)	0.772	0.773	0.727 (0.083–6.352)	0.518	0.471 (0.048–4.622)
	CC + CT	71 (41.5%)	8 (38.1%)	—	—	—	—	—
	TT	100 (58.5%)	13 (61.9%)	0.763	0.764	1.154 (0.454–2.9229)	0.682	1.235 (0.450–3.391)
	C	77 (22.5%)	9 (21.4%)	—	—	—	—	—
	T	265 (77.5%)	33 78.6%)	0.873	0.873	1.065 (0.489–2.323)
	**Male**	—	—	—	—	—	—	—
	C	64 (26.4%)	9 (25%)	—	—	—	—	—
	T	178 (73.6%)	27 (75%)	0.854	0.857	1.079 (0.481–2.417)	—	—

The significant *p* values are in bold, N (%): number (percentage). Abbreviations: ACE, Angiotensin-converting enzyme; I, insertion; D, deletion; OR, odds ratio; CI, confidence interval; L, lower; U, upper; HTN, hypertension; DM, diabetes mellitus; CVD, cardiovascular disease.

#### 3.3.4 Clinical laboratory data

##### 3.3.4.1 C-reactive protein

C-reactive protein (CRP) increased along with COVID-19 severity, as shown in Supplementary Tables S6-S8. The mean ± SD serum levels of CRP in intubated patients with DD and II + ID genotypes were 16.057 ± 7.69 and 23.91 ± 8.05, respectively. Also, it was observed that the mean ± SD serum level of CRP in the intubated groups with DD genotype was significantly lower than in the II + ID genotypes group. Also, the serum level of CRP decreased in the expired group with DD genotype compared to II + ID genotype carriers.

In comparing COVID-19 patients with the control group, the increase in the serum level of CRP in the ACE2 TT genotype was significant compared to CC + CT genotypes (12.05 ± 11.39 vs 3.14 ± 1.69; *p* = 0.029).

##### 3.3.4.2 Interleukin-6

In all carriers of genotypes ACE1 rs1799752 and ACE2 rs1978124, the amount of IL-6 was noticeably increased in the control group compared with the COVID-19 group, as well as outpatients compared with inpatients, and in the survived compared with the expired (Supplementary Tables S9-S11). The IL-6 serum levels in patients admitted to the ICU as opposed to those admitted to the non-ICU ward were considerably increased only in groups with ID, ID + II, and ID + DD genotypes. This finding was also detected when comparing intubated with non-intubated patients. The serum level of IL-6 in all rs1978124 genotypes increased with disease severity, except for the CC genotype.

##### 3.3.4.3 ACE1

The mean serum ACE1 level was significantly higher amongst DD and ID genotypes compared to II genotype in control, COVID-19, outpatients, inpatients, and survived groups. However, this association between ACE1 genotypes and serum ACE1 levels was not observed in ICU, intubated, and expired groups. Even in these groups, the level of ACE1 in the DD genotype was lower than II + ID. Notably, the serum level of ACE1 was significantly decreased in the intubated patients with DD genotype compared to the non-intubated patients with DD genotype. In addition, serum levels of ACE1 in patients with II or II + ID genotypes were increased in both intubated and expired groups compared to non-intubated and survived groups. Supplementary Table S12-S14 summarize the relationship between the serum level of ACE1 and COVID-19 severity.

## 4 Discussion

In this case-control study, the ACE1 I/D and ACE2 (rs1978124) genotypes were found to be distributed in accordance with the Hardy-Weinberg equilibrium, indicating that the chosen samples were representative of the society. This study is the first to evaluate the possible associations between these genetic factors (ACE1 rs1799752 and ACE2 rs1978124) and COVID-19 severity in several major cities in Iran.

The present study showed that HTN, DM, CVD, and renal disease were significantly associated with COVID-19 infection. Also, HTN, DM, and CVD were significantly correlated with the COVID-19 mortality rate. These findings confirm the results of previous studies (Docherty et al., 2020; Ejaz et al., 2020; Garg et al., 2020; Richardson et al., 2020).

Since ACE2 is the cellular receptor for SARS-COV-2 entry and a main component of the RAAS system, the downregulation of ACE2 expression following a viral infection promotes ACE1/ACE2 imbalance (Beyerstedt et al., 2021b). Therefore, studying ACE1 and ACE2 polymorphisms promises to be an effective way of regulating RAAS activity, which can improve the prognosis of COVID-19 patients. Accordingly, numerous studies have been conducted on the association of ACE1 I/D (rs1799752) and ACE2 rs1978124 polymorphisms with different diseases (Rigat et al., 1990; Tiret et al., 1992; Danser et al., 1995; Luo et al., 2019). However, the location of the ACE1 I/D rs1799752 polymorphism and ACE2 rs1978124 SNP in a non-coding region of the genes means they are unlikely to be functional variants. But previous studies have pointed to an association between serum and tissue levels of the ACE1 protein with rs1799752 polymorphism, which can affect the balance of ACE1/ACE2. The rs1799752 polymorphism can describe approximately 50% of ACE activity (Sayed-Tabatabaei et al., 2006). Higher activity of ACE can lead to an increase in the concentration of angiotensin II, which plays an essential role in inflammation (Osadnik et al., 2016).

A recent study showed the effect of a family history of hypertension and ACE1 I/D polymorphism (rs1799752) on cardiac autonomic modulation in adolescents. The D allele is a prognostic factor associated with increased serum ACE levels (Dias-Filho et al., 2021). Another study showed no association between the rs1799752 ACE I/D polymorphism and diabetic retinopathy (Li et al., 2013). The ACE2 rs1978124 SNP is a common genetic factor for cardiovascular disease (Yang et al., 2006; Palmer et al., 2008; Chaoxin et al., 2013), diabetes (Patel et al., 2012), and hypertension (Benjafield et al., 2004). Since the results of relevant studies in different populations display considerable variation, it is necessary that other ethnic groups should be evaluated separately. The present study showed that ACE1 I/D and ACE2 rs1978124 genotypes/alleles frequencies are not significantly correlated with diabetes mellitus, HTN, CVD, and renal disease.

That CRP and IL-6 levels reflect disease severity (Rostamian et al., 2020; Wang, 2020; Luan et al., 2021; Santa Cruz et al., 2021). Accordingly, we also observed that CRP levels were higher in intubated patients with genotype II + ID compared to those with genotype DD. Also, comparing the two groups of COVID-19 and control in terms of ACE2, the CRP level was found to be significantly higher in carriers of TT genotype, which is positively correlated with COVID-19 susceptibility.

Recent studies have demonstrated that ACE1 I/D polymorphism could have a significant role in the prognosis of COVID-19, but these results are controversial (Delanghe et al., 2020a; Bellone and Calvisi, 2020; Saadat, 2020; Yamamoto et al., 2020; Hubacek et al., 2021). Even though numerous studies have reported that the ACE1 DD genotype is associated with COVID-19 severity, they are mostly limited to epidemiological studies and in silico analyses (Gómez et al., 2020; Pati et al., 2020; Verma et al., 2021). Also, a study on the population of Turkey indicated that ACE1 I/D polymorphism was not associated with the severity of COVID-19 (Karakaş Çelik et al., 2021). Overall, we found no significant relationship between ACE1 I/D genotypes and the susceptibility to COVID-19.

Interestingly, we found that the frequencies of DD genotype and D allele were significantly low in the group of patients admitted to the ICU, intubated, and expired. Notably, this association remained significant, even after adjustment. Our results are consistent with the findings of Delanghe et al. They found a significant inverse association between the frequency of the D allele and mortality rate in a study that spanned more than 25 countries (Delanghe et al., 2020b; Delanghe et al., 2020c). Saad H et al. found a positive correlation between ACE1 II genotype and a heightened risk of contracting COVID-19 (Saad et al., 2021). In addition, Hubacek JA et al. showed that ACE1 II genotypes increased the risk of symptomatic COVID-19 in the Czech Republic (Hubacek et al., 2021). Furthermore, Jacobs found a significant elevation in the level of ACE2 protein in the alveolar epithelium cells when patients had II genotype of the rs1799752 polymorphism, as it can facilitate host cell entry of SARS-CoV-2 (Jacobs et al., 2021).

In the COVID-19 outpatients and healthy control, the serum level of ACE1 was significantly higher in DD compared with II + ID genotypes carriers, as expected. Furthermore, the ACE1 level was considerably higher in COVID-19 outpatients than in control subjects. Nevertheless, this significant association was not seen in patients admitted to ICU, intubated, and expired compared to those admitted to the non-ICU ward, not intubated, and who survived. In fact, in these groups, the ACE1 level had even decreased among the DD genotype and increased among II + ID genotypes. Therefore, the serum ACE1 level can be an influential factor in disease prognosis. Similar to our results, Annoni F et al. demonstrated that ACE1 levels were higher in non-survivors compared with survivors of ARDS (Annoni et al., 2019). Therefore, elevated ACE1 levels are associated with poor prognosis in ARDS patients (Sriram and Insel, 2020), which might point to endothelial activation and can prove to be a therapeutic target. Cambien F et al. demonstrated that plasma level of ACE was higher in myocardial infarction patients compared with the control group among subjects with II and ID genotypes (Cambien et al., 1994).

In conclusion, we observed that the ACE I/D polymorphism might alter ACE, IL-6, and CRP expression levels.

Previous studies have demonstrated a relation between ACE2 rs1978124 gene polymorphism and underlying comorbidities affecting the severity of COVID-19. A case-control study was conducted in West China to evaluate the association of rs1978124 ACE2 polymorphism with diabetes. It was revealed that there is found a significant relationship between the frequency of TT + CT genotypes and diabetes (OR = 2.2) (Liu et al., 2018). Another study in China showed a significant relationship between the TT + CT genotypes of rs1978124 polymorphism and dyslipidemia (Pan et al., 2018). Barry R. Palmer also found that the T allele of the ACE2 SNP rs1978124 was associated with higher mortality in an acute coronary syndrome cohort of European ancestry (Palmer et al., 2008).

A recent study on 318 Spanish COVID-19 patients aimed to evaluate the association of rs1978124 polymorphism with the severity of the disease. This study showed that CT genotype in women has a protective role (OR = 0.32) against the severity of COVID-19 (Sabater Molina et al., 2022). Our results also showed that the TT + CT genotypes have a protective effect (OR = 0.098) against the severity of COVID-19 in females. However, we observed that TT + CT genotypes in females have a significant positive role in the susceptibility to COVID-19 (infectivity), and even after adjusting for age and underlying diseases, this association remained significant. Also, the serum level of CRP was higher in patients with TT + CT genotypes compared to the control group. The functional mechanism by which rs1978124 SNP, a noncoding region of the ACE2 gene, affects the outcome of COVID-19 is unclear and needs further investigation. One possible explanation is that polymorphism affects the stability of ACE2 mRNA (i.e., splicing), post-transcriptional regulation by microRNA, and the efficiency of mRNA splicing (for example, silencing element or enhancer of intron splicing).

## 5 Study limitations

One of the limitations of this study was that the blood sample volume obtained from some patients was insufficient for laboratory tests, such as the ACE1, CRP, and IL-6 serum levels tests. Moreover, even though the assessment of angiotensin II and ACE2 serum levels can provide additional evidence for predicting the outcome of COVID-19, unfortunately, no blood sample was left to perform these tests.

## 6 Conclusion

We was demonstrated that the ACE1 I/D and ACE2 rs1978124 polymorphisms are relevant prognostic factors for the outcome of COVID-19. Patients with the II + ID genotype might have a significantly worse prognosis than those with the DD genotype. The T allele of SNP rs1978124 could affect susceptibility to COVID-19; ACE1/ACE2 polymorphism-mediated pathology is relevant, at least in the Iranian population. Consequently, further genetic studies on COVID-19 patients in different countries are required to clarify the mechanisms of lung injury and determine new therapeutic approaches.

## Data Availability

The datasets presented in this study can be found in online repositories. The names of the repository/repositories and accession number(s) can be found in the article/Supplementary Material.
