# Supplementary material for: The role of ACE1 I/D and ACE2 polymorphism in the outcome of Iranian COVID-19 patients: A case-control study
Source: Front Genet. 2022 Sep 5;13:955965. doi: 10.3389/fgene.2022.955965 (PMC9483011; doi:10.3389/fgene.2022.955965)
Supplement: Supplementary file 1 [file DataSheet1.docx]

Supplementary Table S1: Association between Demographic Characteristics and Severity of COVID-19.

|  | Control  (n=56) | Outpatient  (n=207) | Inpatient  (n=263) | P-value | Inpatient (n=207) | | | | | | COVID-19 (n=470) | | |
| --- | --- | --- | --- | --- | --- | --- | --- | --- | --- | --- | --- | --- | --- |
|  |  |  |  |  | ICU | | P-value | Intubation | | P-value | Survived  (n=413) | Expired  (n=57) | P-value |
|  |  |  |  |  | No (n=187) | Yes (n=76) |  | No (n=216) | Yes  (n=47) |  |  |  |  |
| sex |  |  |  |  |  |  |  |  |  |  |  |  |  |
| Female | 31  (55.4%) | 83  (40.1%) | 109  (41.4%) |  | 78  (41.7%) | 31  (40.8%) |  | 91  (42.1%) | 18  (38.3%) |  | 171  (41.4%) | 21  (36.8%) |  |
| Male | 25  (44.6%) | 124  (59.9%) | 154  (58.6%) | 0.111 | 109  (58.3%) | 45  (59.2%) | 0.891 | 125  (57.9%) | 29  (61.7%) | 0.629 | 242  (58.6%) | 36  (63.2%) | 0.511 |
| cigarette smoking |  |  |  |  |  |  |  |  |  |  |  |  |  |
| No | 54  (96.4%) | 197  (95.2%) | 238  (90.5%) |  | 170  (90.9%) | 68  (89.5%) |  | 195  (90.3%) | 43  (91.5%) |  | 382  (92.5%) | 53  (93.0%) |  |
| Yes | 2  (3.6%) | 10  (4.8%) | 25  (9.5%) | 0.081 | 17  (9.1%) | 8  (10.5%) | 0.719 | 21  (9.7%) | 4  (8.5%) | 0.797 | 31  (7.5%) | 4  (7.0%) | 0.895 |
| HTN |  |  |  |  |  |  |  |  |  |  |  |  |  |
| No | 54  (96.4%) | 183  (88.4%) | 158  (60.1%) |  | 112  (59.9%) | 46  (60.5%) |  | 128  (59.3%) | 30  (63.8%) |  | 308  (74.6%) | 33  (57.9%) |  |
| Yes | 2  (3.6%) | 24  (11.6%) | 105  (39.9%) | **<0.001** | 75  (40.1%) | 30  (39.5%) | 0.924 | 88  (40.7%) | 17  (36.2%) | 0.564 | 105  (25.4%) | 24  (42.1%) | **0.008** |
| DM |  |  |  |  |  |  |  |  |  |  |  |  |  |
| No | 55  (98.2%) | 191  (92.3%) | 179  (68.1%) |  | 129  (69.0%) | 50  (65.8%) |  | 149  (69.0%) | 30  (63.8%) |  | 333  (80.6%) | 37  (64.9%) |  |
| Yes | 1  (1.8%) | 16  (7.7%) | 84  (31.9%) | **<0.001** | 58  (31.0%) | 26  (34.2%) | 0.614 | 67  (31.0%) | 17  (36.2%) | 0.492 | 80  (19.4%) | 20  (35.1%) | **0.007** |
| CVD |  |  |  |  |  |  |  |  |  |  |  |  |  |
| No | 56  (100.0%) | 202  (97.6%) | 186  (70.7%) |  | 134  (71.7%) | 52  (68.4%) |  | 152  (70.4%) | 34  (72.3%) |  | 348  (84.3%) | 40  (70.2%) |  |
| Yes | 0  (0.0%) | 5  (2.4%) | 77  (29.3%) | **<0.001** | 53  (28.3%) | 24  (31.6%) | 0.601 | 64  (29.6%) | 13  (27.7%) | 0.778 | 65  (15.7%) | 17  (29.8%) | **0.009** |
| Renal disease |  |  |  |  |  |  |  |  |  |  |  |  |  |
| No | 56  (100.0%) | 199  (96.1%) | 242  (92.0%) |  | 175  (93.6%) | 67  (88.2%) |  | 200  (92.6%) | 42  (89.4%) |  | 390  (94.4%) | 51  (89.5%) |  |
| Yes | 0  (0.0%) | 8  (3.9%) | 21  (8.0%) | **0.024** | 12  (6.4%) | 9  (11.8%) | 0.141 | 16  (7.4%) | 5  (10.6%) | 0.459 | 23  (5.6%) | 6  (10.5%) | 0.145 |

ACE: Angiotensin-converting enzyme; ICU: Intensive care unit; HTN: Hypertension; DM: Diabetes mellitus; CVD: Cardiovascular disease; COVID-19: Coronavirus disease 2019.

Supplementary Table S2: Hardy-Weinberg Equilibrium.

|  | | **Control** | | **COVID-19** | | **Outpatient** | | **Inpatient** | |
| --- | --- | --- | --- | --- | --- | --- | --- | --- | --- |
|  |  | Observed | Expected | Observed | Expected | Observed | Expected | Observed | Expected |
| **ACE1 I/D** | II | 9 | 10.3 | 107 | 97.9 | 44 | 39.6 | 63 | 58.5 |
|  | ID | 30 | 27.4 | 215 | 233.2 | 93 | 101.9 | 122 | 131.1 |
|  | DD | 17 | 18.3 | 148 | 138.9 | 70 | 65.6 | 78 | 73.5 |
|  | P | 0.429 | | 0.456 | | 0.437 | | 0.471 | |
|  | q | 0.571 | | 0.544 | | 0.563 | | 0.529 | |
|  | x2 | 0.492 | | 2.866 | | 1.568 | | 1.260 | |
|  | P-value | 0.483 | | 0.090 | | 0.210 | | 0.262 | |
| **ACE2 rs1978124** | CC | 12 | 6.4 | 80 | 28.6 | 33 | 12.1 | 47 | 16.6 |
|  | CT | 14 | 25.1 | 72 | 174.7 | 34 | 75.8 | 38 | 98.9 |
|  | TT | 30 | 24.4 | 318 | 266.6 | 140 | 119.1 | 178 | 147.6 |
|  | P | 0.339 | | 0.247 | | 0.242 | | 0.251 | |
|  | q | 0.661 | | 0.753 | | 0.758 | | 0.749 | |
|  | x2 | 10.960 | | 162.478 | | 63.010 | | 99.691 | |
|  | P-value | **0.001** | | **<0.001** | | **<0.001** | | **<0.001** | |

Supplementary Table S3: Association Genotype Frequencies of the ACE1 Polymorphism with HTN, DM, CVD, and renal disease of Individuals In COVID-19 Patients.

|  | | **HTN** | | | **DM** | | | **CVD** | | | **Renal disease** | | | |
| --- | --- | --- | --- | --- | --- | --- | --- | --- | --- | --- | --- | --- | --- | --- |
|  |  | No | Yes | P-value | No | Yes | P-value | No | Yes | P-value | No | Yes | P-value |  |
| **ACE1 I/D** | II | 81 (23.8%) | 26 (20.2%) |  | 79 (21.4%) | 28 (28.0%) |  | 92 (23.7%) | 15 (18.3%) |  | 98 (22.2%) | 9 (31.0%) |  |  |
|  | ID | 150 (44.0%) | 65 (50.4%) | 0.449 | 172 (46.5%) | 43 (43.0%) | 0.370 | 174 (44.8%) | 41 (50.0%) | 0.531 | 201 (45.6%) | 14 (48.3%) | 0.347 |  |
|  | DD | 110 (32.3%) | 38 (29.5%) |  | 119 (32.2%) | 29 (29.0%) |  | 122 (31.4%) | 26 (31.7%) |  | 142 (32.2%) | 6 (20.7%) |  |  |
|  | II | 81 (23.8%) | 26 (20.2%) |  | 79 (21.4%) | 28 (28.0%) |  | 92 (23.7%) | 15 (18.3%) |  | 98 (22.2%) | 9 (31.0%) |  |  |
|  | ID + DD | 260 (76.2%) | 103 (79.8%) | 0.406 | 291 (78.6%) | 72 (72.0%) | 0.159 | 296 (76.3%) | 67 (81.7%) | 0.288 | 343 (77.8%) | 20 (69.0%) | 0.273 |  |
|  | II + ID | 231 (67.7%) | 91 (70.5%) |  | 251 (67.8%) | 71 (71.0%) |  | 266 (68.6%) | 56 (68.3%) |  | 299 (67.8%) | 23 (79.3%) |  |  |
|  | DD | 110 (32.3%) | 38 (29.5%) | 0.560 | 119 (32.2%) | 29 (29.0%) | 0.546 | 122 (31.4%) | 26 (31.7%) | 0.963 | 142 (32.2%) | 6 (20.7%) | 0.196 |  |
|  | I | 312 (45.7%) | 117 (45.3%) |  | 330 (44.6%) | 99 (49.5%) |  | 358 (46.1%) | 71 (43.3%) |  | 397 (45.0%) | 32 (55.2%) |  |  |
|  | D | 370 (54.3%) | 141 (54.7%) | 0.913 | 410 (55.4%) | 101 (50.5%) | 0.230 | 418 (53.9%) | 93 (56.7%) | 0.507 | 485 (55.0%) | 26 (44.8%) | 0.132 |  |
| **ACE2 rs1978124** | **Female** |  |  |  |  |  |  |  |  |  |  |  |  |  |
|  | CC | 6 (4.6%) | 1 (1.6%) |  | 6 (4.3%) | 1 (2.0%) |  | 6 (3.8%) | 1 (2.8%) |  | 6 (3.3%) | 1 (9.1%) |  |  |
|  | CT | 48 (36.9%) | 24 (38.7%) | 0.582 | 55 (39.0%) | 17 (33.3%) | 0.531 | 56 (35.9%) | 16 (44.4%) | 0.625 | 67 (37.0%) | 5 (45.5%) | 0.472 |  |
|  | TT | 76 (58.5%) | 37 (59.7%) |  | 80 (56.7%) | 33 (64.7%) |  | 94 (60.3%) | 19 (52.8%) |  | 108 (59.7%) | 5 (45.5%) |  |  |
|  | CC | 6 (4.6%) | 1 (1.6%) |  | 6 (4.3%) | 1 (2.0%) |  | 6 (3.8%) | 1 (2.8%) |  | 6 (3.3%) | 1 (9.1%) |  |  |
|  | TT + CT | 124 (95.4%) | 61 (98.4%) | 0.299 | 135 (95.7%) | 50 (98.0%) | 0.454 | 150 (96.2%) | 35 (97.2%) | 0.758 | 175 (96.7%) | 10 (90.9%) | 0.321 |  |
|  | CC + CT | 54 (41.5%) | 25 (40.3%) |  | 61 (43.3%) | 18 (35.3%) |  | 62 (39.7%) | 17 (47.2%) |  | 73 (40.3%) | 6 (54.5%) |  |  |
|  | TT | 76 (58.5%) | 37 (59.7%) | 0.873 | 80 (56.7%) | 33 (64.7%) | 0.446 | 94 (60.3%) | 19 (52.8%) | 0.411 | 108 (59.7%) | 5 (45.5%) | 0.352 |  |
|  | C | 60 (23.1%) | 26 (21.0%) |  | 67 (23.8%) | 19 (18.6%) |  | 68 (21.8%) | 18 (25.0%) |  | 79 (21.8%) | 7 (31.8%) |  |  |
|  | T | 200 (76.9%) | 98 (79.0%) | 0.643 | 215 (76.2%) | 83 (81.4%) | 0.287 | 244 (78.2%) | 54 (75.0%) | 0.557 | 283 (78.2%) | 15 (68.2%) | 0.275 |  |
|  | **Male** |  |  |  |  |  |  |  |  |  |  |  |  |  |
|  | C | 65 (25%) | 15 (22.4%) |  | 62 (27.1%) | 11 (22.4%) |  | 66 (28.4%) | 7 (15.2%) |  | 65 (25%) | 8 (44.4%) |  |  |
|  | T | 195 (75%) | 52 (77.6%) | 0.490 | 167 (72.9%) | 38 (77.6%) | 0.504 | 166 (71.6%) | 39 (84.8%) | 0.062 | 195 (75%) | 10 (55.6%) | 0.070 |  |

ACE: Angiotensin-converting enzyme; HTN: Hypertension; DM: Diabetes mellitus; CVD: Cardiovascular disease.

Supplementary Table S4: Association ACE1 I/D and ACE2 rs1978124 Genotypes/ Alleles distribution with susceptibility to COVID-19, adjusted by age, sex, Cigarette smoking, alcohol consumption, diabetes, HTN, CVA, and renal diseases.

| Genotypes  Alleles  N (%) | | **Study group** | | | **Unadjusted** | | **Adjusted** | | |
| --- | --- | --- | --- | --- | --- | --- | --- | --- | --- |
|  |  | Outpatients  (n=207) | Inpatients  (n=263) | P-value  (Chi-square) | P-value | OR-95%CI- (L-U) | P-value | OR-95%CI- (L-U) |  |
| **ACE1 I/D** | II | 44 (21.3%) | 63 (24%) |  |  |  |  |  |  |
|  | ID | 93 (44.9%) | 122 (46.4%) | 0.588 | 0.715 | 0.916 (0.573-1.466) | 0.724 | 1.110 (0.621-1.984) |  |
|  | DD | 70 (33.8%) | 78 (29.7%) |  | 0.328 | 0.778 (0.471-1.286) | 0.968 | 1.013 (0.540-1.900) |  |
|  | II | 44 (21.3%) | 63 (24%) |  |  |  |  |  |  |
|  | ID + DD | 163 (78.7%) | 200 (76%) | 0.489 | 0.499 | 0.857 (9.544-1.327) | 0.808 | 1.071 (0.623-1.839) |  |
|  | II + ID | 137 (66.2%) | 185 (70.3%) |  |  |  |  |  |  |
|  | DD | 70 (33.8%) | 78 (29.7%) | 0.335 | 0.336 | 0.825 (0.558-1.220) | 0.821 | 0.994 (0.576-1.548) |  |
|  | I | 181 (43.7%) | 248 (47.1%) |  |  |  |  |  |  |
|  | D | 233 (61.8%) | 278 (52.9%) | 0.295 | 0.295 | 0.871 (0.672-1.128) |  |  |  |
| **ACE2 rs1978124** | **Female** |  |  |  |  |  |  |  |  |
|  | CC | 1 (1.2%) | 6 (5.5%) |  |  |  |  |  |  |
|  | CT | 34 (41%) | 38 (34.9%) | 0.237 | 0.129 | 0.186 (0.021-1.627) | **0.022** | 0.069 (0.007-0.684) |  |
|  | TT | 48 (57.8%) | 65 (59.6%) |  | 0.175 | 0.226 (0.026-1.937) | 0.065 | 0.120 (0.013-1.140) |  |
|  | CC | 1 (1.2%) | 6 (5.5%) |  |  |  |  |  |  |
|  | TT + CT | 82 (98.8%) | 103 (94.5%) | 0.151 | 0.151 | 0.209 (0.025-1.774) | **0.042** | 0.099 (0.011-0.920) |  |
|  | CC + CT | 35 (42.2%) | 44 (40.4%) |  |  |  |  |  |  |
|  | TT | 48 (57.8%) | 65 (59.6%) | 0.802 | 0.802 | 1.077 (0.603-1.924) | 0.506 | 1.291 (0.607-2.747) |  |
|  | C | 36 (21.7%) | 50 (22.9%) |  |  |  |  |  |  |
|  | T | 130 (69.9%) | 168 (77.1%) | 0.771 | 0.771 | 0.930 (0.572-1.512) |  |  |  |
|  | **Male** |  |  |  |  |  |  |  |  |
|  | C | 32 (25.8%) | 41 (26.6%) |  |  |  |  |  |  |
|  | T | 92 (74.2%) | 113 (73.4%) | 0.878 | 0.880 | 0.959 (0.560-1.642) |  |  |  |

Supplementary Table S5: Association of ACE1 I/D and ACE2 rs1978124 Genotypes/ Alleles Distribution with COVID-19 Severity, Adjusted by Age, Sex, Cigarette Smoking, DM, HTN, CVD, and renal diseases

| Genotypes  Alleles  N (%) | | **Inpatients ICU Admitted** | | | **Unadjusted** | | **Adjusted** | | |
| --- | --- | --- | --- | --- | --- | --- | --- | --- | --- |
|  |  | No  (n=187) | Yes  (n=76) | P-value  (Chi-square) | P-value | OR-95%CI- (L-U) | P-value | OR-95%CI- (L-U) |  |
| **ACE1 I/D** | II | 42 (22.5%) | 21 (27.6%) |  |  |  |  |  |  |
|  | ID | 83 (44.4%) | 39 (51.3%) | 0.147 | 0.088 | 1.937 (0.907-4.140) | 0.942 | 1.025 (0.520-2.023) |  |
|  | DD | 62 (33.2%) | 16 (21.1%) |  | 0.079 | 1.821 (0.933-3.553) | 0.113 | 0.533 (0.244-1.161) |  |
|  | II | 42 (22.5%) | 21 (27.6%) |  |  |  |  |  |  |
|  | ID + DD | 145 (77.5%) | 55 (72.4%) | 0.373 | 0.374 | 0.759 (0.413-1.395) | 0.489 | 0.800 (0.425-1.506) |  |
|  | II + ID | 125 (66.8%) | 60 (78.9%) |  |  |  |  |  |  |
|  | DD | 62 (33.2%) | 16 (21.1%) | 0.051 | 0.053 | 0.538 (0.286-1.009) | **0.049** | 0.534 (0.275-0.998) |  |
|  | I | 167 (44.7%) | 81 (53.3%) |  |  |  |  |  |  |
|  | D | 207 (61%) | 71 (46.7%) | 0.072 | 0.073 | 0.707 (0.484-1.032) |  |  |  |
| **ACE2 rs1978124** | **Female** |  |  |  |  |  |  |  |  |
|  | CC | 5 (6.4%) | 1 (3.2%) |  |  |  |  |  |  |
|  | CT | 24 (30.8%) | 14 (45.2%) | 0.334 | 0.350 | 2.917 (0.309-27.560) | 0.543 | 2.095 (0.193-22.763) |  |
|  | TT | 49 (62.8%) | 16 (51.6%) |  | 0.655 | 1.633 (0.177-15.032) | 0.830 | 1.297 (0.121-13.902) |  |
|  | CC | 5 (6.4%) | 1 (3.2%) |  |  |  |  |  |  |
|  | TT + CT | 73 (93.6%) | 30 (96.8%) | 0.511 | 0.519 | 2.055 (0.230-18.336) | 0.694 | 1.601 (0.154-16.632) |  |
|  | CC + CT | 29 (37.2%) | 15 (48.4%) |  |  |  |  |  |  |
|  | TT | 49 (62.8%) | 16 (51.6%) | 0.282 | 0.282 | 0.631 (0.272-1.464) | 0.382 | 0.665 (0.267-1.658) |  |
|  | C | 34 (21.8%) | 16 (25.8%) |  |  |  |  |  |  |
|  | T | 122 (62.2%) | 46 (74.2%) | 0.525 | 0.526 | 0.801 (0.404-1.588) |  |  |  |
|  | **Male** |  |  |  |  |  |  |  |  |
|  | C | 28 (25.7%) | 13 (28.9%) |  |  |  |  |  |  |
|  | T | 81 (74.3%) | 32 (71.1%) | 0.683 | 0.684 | 0.851 (0.392-1.915) |  |  |  |

The significant P values are in bold, n (%): number (percentage). Abbreviations: ACE, Angiotensin-converting enzyme; I, insertion; D, deletion; OR, odds ratio; CI, confidence interval; L, lower; U, upper; ICU, Intensive Care Unit; HTN, hypertension; DM, diabetes mellitus; CVD, cardiovascular disease.

Supplementary Table S6: Association ACE1 I/D and ACE2 rs1978124 Genotypes/ Alleles Distribution with Serum CRP Levels.

| Genotypes  Alleles | | CRP (U/L) | | | | | | | | | | | | | |
| --- | --- | --- | --- | --- | --- | --- | --- | --- | --- | --- | --- | --- | --- | --- | --- |
|  |  | Control | | | COVID-19 | | | P-value | Outpatient | | | Inpatient | | | P-value |
|  |  | N | Mean | SD | N | Mean | SD |  | N | Mean | SD | N | Mean | SD |  |
| **ACE1 I/D** | II | 9 | 3.51 | 1.92 | 98 | 13.18 | 11.35 | **0.040** | 41 | 3.48 | 4.44 | 57 | 20.15 | 9.52 | **<0.001** |
|  | ID | 30 | 3.57 | 1.61 | 198 | 13.08 | 11.62 | **0.003** | 84 | 3.53 | 3.97 | 114 | 20.11 | 10.29 | **<0.001** |
|  | DD | 17 | 2.69 | 1.20 | 134 | 10.62 | 10.10 | **0.003** | 64 | 4.02 | 4.86 | 70 | 16.65 | 9.89 | **<0.001** |
|  | P-value |  | 0.249 |  |  | 0.287 |  |  |  | 0.790 |  |  | 0.078 |  |  |
|  | II | 9 | 3.51 | 1.92 | 98 | 13.18 | 11.35 | **0.040** | 41 | 3.48 | 4.44 | 57 | 20.15 | 9.52 | **<0.001** |
|  | ID + DD | 47 | 3.25 | 1.52 | 332 | 12.08 | 11.08 | **<0.001** | 148 | 3.74 | 4.37 | 184 | 18.80 | 10.25 | **<0.001** |
|  | P-value |  | 0.647 |  |  | 0.399 |  |  |  | 0.657 |  |  | 0.386 |  |  |
|  | II + ID | 39 | 3.56 | 1.66 | 296 | 13.11 | 11.51 | **<0.001** | 125 | 3.51 | 4.11 | 171 | 20.13 | 10.01 | **<0.001** |
|  | DD | 17 | 2.69 | 1.20 | 134 | 10.62 | 10.10 | **0.003** | 64 | 4.02 | 4.86 | 70 | 16.65 | 9.89 | **<0.001** |
|  | P-value |  | 0.095 |  |  | 0.121 |  |  |  | 0.514 |  |  | **0.024** |  |  |
| **ACE2 rs1978124** | **Female** |  |  |  |  |  |  |  |  |  |  |  |  |  |  |
|  | CC | 6 | 3.38 | 1.72 | 7 | 15.53 | 11.06 | 0.086 | 1 | 1.00 | . | 6 | 17.95 | 9.87 | 0.134 |
|  | CT | 14 | 4.22 | 1.44 | 70 | 10.12 | 10.82 | 0.900 | 32 | 2.84 | 2.91 | 38 | 16.25 | 11.26 | **<0.001** |
|  | TT | 11 | 3.14 | 1.69 | 104 | 12.05 | 11.39 | **0.029** | 45 | 3.99 | 4.78 | 59 | 18.20 | 11.15 | **<0.001** |
|  | P-value |  | 0.216 |  |  | 0.328 |  |  |  | 0.185 |  |  | 0.842 |  |  |
|  | CC | 6 | 3.38 | 1.72 | 7 | 15.53 | 11.06 | 0.086 | 1 | 1.00 | . | 6 | 17.95 | 9.87 | 0.134 |
|  | TT + CT | 25 | 3.74 | 1.62 | 174 | 11.27 | 11.17 | 0.076 | 77 | 3.51 | 4.12 | 97 | 17.44 | 11.18 | **<0.001** |
|  | P-value |  | 0.510 |  |  | 0.356 |  |  |  | 0.142 |  |  | 0.944 |  |  |
|  | CC + CT | 20 | 3.97 | 1.54 | 77 | 10.61 | 10.88 | 0.456 | 33 | 2.78 | 2.88 | 44 | 16.48 | 10.99 | **<0.001** |
|  | TT | 11 | 3.14 | 1.69 | 104 | 12.05 | 11.39 | **0.029** | 45 | 3.99 | 4.78 | 59 | 18.20 | 11.15 | **<0.001** |
|  | P-value |  | 0.215 |  |  | 0.355 |  |  |  | 0.197 |  |  | 0.591 |  |  |
|  | **Male** |  |  |  |  |  |  |  |  |  |  |  |  |  |  |
|  | C | 6 | 3.58 | 1.56 | 68 | 13.19 | 11.25 | 0.148 | 31 | 3.43 | 4.30 | 37 | 21.37 | 8.33 | **<0.001** |
|  | T | 19 | 2.58 | 1.33 | 181 | 12.90 | 11.06 | **<0.001** | 80 | 3.98 | 4.68 | 101 | 19.97 | 9.42 | **<0.001** |
|  | P-value |  | 0.264 |  |  | 0.946 |  |  |  | 0.569 |  |  | 0.757 |  |  |

ACE: Angiotensin-converting enzyme; CRP: C-Reactive Protein.

Supplementary Table S7: Association ACE1 I/D and ACE2 rs1978124 Genotypes/ Alleles Distribution with Serum CRP Levels.

| Genotypes  Alleles | | CRP (U/L) | | | | | | | | | | | | | |
| --- | --- | --- | --- | --- | --- | --- | --- | --- | --- | --- | --- | --- | --- | --- | --- |
|  |  | Inpatient  ICU-No | | | Inpatient  ICU-Yes | | | P-value | Inpatient  Intubated -No | | | Inpatient  Intubated -Yes | | | P-value |
|  |  | N | Mean | SD | N | Mean | SD |  | N | Mean | SD | N | Mean | SD |  |
| **ACE1 I/D** | II | 40 | 18.19 | 10.17 | 17 | 24.77 | 5.71 | 0.035 | 46 | 18.60 | 9.68 | 11 | 26.65 | 5.35 | **0.015** |
|  | ID | 78 | 19.26 | 10.84 | 36 | 21.96 | 8.85 | 0.206 | 90 | 19.44 | 10.59 | 24 | 22.66 | 8.84 | 0.178 |
|  | DD | 56 | 16.42 | 9.93 | 14 | 17.57 | 10.06 | 0.660 | 64 | 16.65 | 10.13 | 6 | 16.57 | 7.69 | 0.941 |
|  | P-value |  | 0.360 |  |  | 0.212 |  |  |  | 0.359 |  |  | 0.075 |  |  |
|  | II | 40 | 18.19 | 10.17 | 17 | 24.77 | 5.71 | 0.035 | 46 | 18.60 | 9.68 | 11 | 26.65 | 5.35 | **0.015** |
|  | ID + DD | 134 | 18.07 | 10.52 | 50 | 20.73 | 9.32 | 0.112 | 154 | 18.28 | 10.45 | 30 | 21.44 | 8.85 | 0.140 |
|  | P-value |  | 0.852 |  |  | 0.234 |  |  |  | 0.865 |  |  | 0.112 |  |  |
|  | II + ID | 118 | 18.90 | 10.58 | 53 | 22.86 | 8.03 | 0.030 | 136 | 19.15 | 10.26 | 35 | 23.91 | 8.05 | **0.014** |
|  | DD | 56 | 16.42 | 9.93 | 14 | 17.57 | 10.06 | 0.660 | 64 | 16.65 | 10.13 | 6 | 16.57 | 7.69 | 0.941 |
|  | P-value |  | 0.166 |  |  | 0.110 |  |  |  | 0.167 |  |  | 0.048 |  |  |
| **ACE2 rs1978124** | **Female** |  |  |  |  |  |  |  |  |  |  |  |  |  |  |
|  | CC | 5 | 18.52 | 10.93 | 1 | 15.10 | . | 0.770 | 5 | 18.52 | 10.93 | 1 | 15.10 | . | 0.770 |
|  | CT | 24 | 14.08 | 10.80 | 14 | 19.97 | 11.44 | 0.119 | 30 | 13.82 | 10.69 | 8 | 25.39 | 8.72 | **0.009** |
|  | TT | 45 | 18.01 | 11.61 | 14 | 18.81 | 9.89 | 0.702 | 51 | 18.07 | 11.48 | 8 | 19.04 | 9.40 | 0.816 |
|  | P-value |  | 0.437 |  |  | 0.663 |  |  |  | 0.320 |  |  | 0.156 |  |  |
|  | CC | 5 | 18.52 | 10.93 | 1 | 15.10 | . | 0.770 | 5 | 18.52 | 10.93 | 1 | 15.10 | . | 0.770 |
|  | TT + CT | 69 | 16.64 | 11.41 | 28 | 19.39 | 10.51 | 0.185 | 81 | 16.49 | 11.31 | 16 | 22.21 | 9.35 | **0.035** |
|  | P-value |  | 0.707 |  |  | 0.473 |  |  |  | 0.664 |  |  | 0.307 |  |  |
|  | CC + CT | 29 | 14.85 | 10.76 | 15 | 19.65 | 11.09 | 0.158 | 35 | 14.49 | 10.69 | 9 | 24.24 | 8.84 | **0.017** |
|  | TT | 45 | 18.01 | 11.61 | 14 | 18.81 | 9.89 | 0.702 | 51 | 18.07 | 11.48 | 8 | 19.04 | 9.40 | 0.816 |
|  | P-value |  | 0.308 |  |  | 0.678 |  |  |  | 0.199 |  |  | 0.178 |  |  |
|  | **Male** |  |  |  |  |  |  |  |  |  |  |  |  |  |  |
|  | ‌C | 27 | 19.81 | 8.52 | 10 | 25.59 | 6.38 | 0.108 | 33 | 20.17 | 7.95 | 4 | 31.28 | 3.23 | **0.007** |
|  | T | 73 | 18.82 | 10.06 | 28 | 23.00 | 6.78 | 0.200 | 81 | 19.47 | 9.82 | 20 | 22.04 | 7.49 | 0.720 |
|  | P-value |  | 0.972 |  |  | 0.320 |  |  |  | 0.731 |  |  | **0.015** |  |  |

ACE: Angiotensin-converting enzyme; CRP: C-Reactive Protein.

Supplementary Table S8: Association ACE1 I/D and ACE2 rs1978124 Genotypes/ Alleles Distribution with Serum CRP Levels.

| Genotypes  Alleles | | CRP (U/L) | | | | | | |
| --- | --- | --- | --- | --- | --- | --- | --- | --- |
|  |  | Survived | | | Deceased | | | P-value |
|  |  | N | Mean | SD | N | Mean | SD |  |
| **ACE1 I/D** | II | 85 | 11.06 | 10.53 | 13 | 27.04 | 4.99 | **<0.001** |
|  | ID | 171 | 11.51 | 11.33 | 27 | 22.99 | 8.08 | **<0.001** |
|  | DD | 126 | 10.09 | 10.05 | 8 | 18.89 | 7.28 | **0.012** |
|  | P-value |  | 0.879 |  |  | 0.057 |  |  |
|  | II | 85 | 11.06 | 10.53 | 13 | 27.04 | 4.99 | **<0.001** |
|  | ID + DD | 297 | 10.91 | 10.81 | 35 | 22.05 | 7.99 | **<0.001** |
|  | P-value |  | 0.814 |  |  | 0.051 |  |  |
|  | II + ID | 256 | 11.36 | 11.05 | 40 | 24.31 | 7.41 | **<0.001** |
|  | DD | 126 | 10.09 | 10.05 | 8 | 18.89 | 7.28 | **0.012** |
|  | P-value |  | 0.613 |  |  | 0.062 |  |  |
| **ACE2 rs1978124** | **Female** |  |  |  |  |  |  |  |
|  | CC | 6 | 15.60 | 12.11 | 1 | 15.10 | . | 1.000 |
|  | CT | 63 | 7.93 | 8.96 | 7 | 29.84 | 3.47 | **<0.001** |
|  | TT | 95 | 11.50 | 11.45 | 9 | 17.89 | 9.45 | 0.091 |
|  | P-value |  | 0.089 |  |  | **0.021** |  |  |
|  | CC | 6 | 15.60 | 12.11 | 1 | 15.10 | . | 1.000 |
|  | TT + CT | 158 | 10.08 | 10.64 | 16 | 23.12 | 9.48 | **<0.001** |
|  | P-value |  | 0.295 |  |  | 0.307 |  |  |
|  | CC + CT | 69 | 8.60 | 9.42 | 8 | 28.00 | 6.12 | **<0.001** |
|  | TT | 95 | 11.50 | 11.45 | 9 | 17.89 | 9.45 | 0.091 |
|  | P-value |  | 0.111 |  |  | **0.027** |  |  |
|  | **Male** |  |  |  |  |  |  |  |
|  | C | 61 | 11.71 | 10.79 | 7 | 26.13 | 5.68 | **0.003** |
|  | T | 157 | 11.34 | 10.77 | 24 | 23.14 | 6.73 | **<0.001** |
|  | P-value |  | 0.991 |  |  | 0.210 |  |  |

ACE: Angiotensin-converting enzyme; CRP: C-Reactive Protein.

Supplementary Table S9: Association ACE1 I/D and ACE2 rs1978124 Genotypes/ Alleles Distribution with Serum IL-6 Levels.

| Genotypes  Alleles | | IL-6 (Ng/L) | | | | | | | | | | | | | |
| --- | --- | --- | --- | --- | --- | --- | --- | --- | --- | --- | --- | --- | --- | --- | --- |
|  |  | Control | | | COVID-19 | | | P-value | Outpatient | | | Inpatient | | | P-value |
|  |  | N | Mean | SD | N | Mean | SD |  | N | Mean | SD | N | Mean | SD |  |
| **ACE1 I/D** | II | 8 | 3.84 | 3.58 | 69 | 57.30 | 174.30 | 0.005 | 25 | 47.51 | 198.68 | 44 | 62.87 | 161.01 | 0.001 |
|  | ID | 24 | 4.32 | 3.69 | 127 | 70.19 | 179.21 | <0.001 | 45 | 34.57 | 150.78 | 82 | 89.74 | 191.09 | <0.001 |
|  | DD | 15 | 4.35 | 4.84 | 92 | 46.88 | 151.51 | 0.004 | 45 | 15.15 | 43.59 | 47 | 77.26 | 204.12 | <0.001 |
|  | P-value |  | 0.727 |  |  | 0.169 |  |  |  | 0.704 |  |  | 0.294 |  |  |
|  | II | 8 | 3.84 | 3.58 | 69 | 57.30 | 174.30 | **0.005** | 25 | 47.51 | 198.68 | 44 | 62.87 | 161.01 | **0.001** |
|  | ID + DD | 39 | 4.33 | 4.11 | 219 | 60.40 | 168.16 | **<0.001** | 90 | 24.86 | 110.79 | 129 | 85.19 | 195.24 | **<0.001** |
|  | P-value |  | 0.560 |  |  | 0.875 |  |  |  | 0.452 |  |  | 0.427 |  |  |
|  | II + ID | 32 | 4.20 | 3.61 | 196 | 65.65 | 177.15 | **<0.001** | 70 | 39.19 | 168.13 | 126 | 80.36 | 180.96 | **<0.001** |
|  | DD | 15 | 4.35 | 4.84 | 92 | 46.88 | 151.51 | **0.004** | 45 | 15.15 | 43.59 | 47 | 77.26 | 204.12 | **<0.001** |
|  | P-value |  | 0.734 |  |  | 0.073 |  |  |  | 0.986 |  |  | 0.329 |  |  |
| **ACE2 rs1978124** | **Female** |  |  |  |  |  |  |  |  |  |  |  |  |  |  |
|  | CC | 4 | 6.32 | 7.38 | 4 | 168.03 | 315.37 | 0.375 | 1 | 2.00 | . | 3 | 223.37 | 361.68 | 0.180 |
|  | CT | 11 | 2.16 | 0.55 | 32 | 52.08 | 175.21 | **<0.001** | 16 | 77.79 | 246.94 | 16 | 26.36 | 32.43 | 0.462 |
|  | TT | 10 | 2.95 | 1.57 | 67 | 53.56 | 179.92 | **0.005** | 28 | 39.90 | 188.22 | 39 | 63.37 | 175.54 | **<0.001** |
|  | P-value |  | 0.177 |  |  | 0.308 |  |  |  | **0.017** |  |  | 0.386 |  |  |
|  | CC | 4 | 6.32 | 7.38 | 4 | 168.03 | 315.37 | 0.375 | 1 | 2.00 | . | 3 | 223.37 | 361.68 | 0.180 |
|  | TT + CT | 21 | 2.54 | 1.19 | 99 | 53.08 | 177.51 | **<0.001** | 44 | 53.68 | 209.42 | 55 | 52.60 | 149.21 | **<0.001** |
|  | P-value |  | 0.166 |  |  | 0.376 |  |  |  | 0.283 |  |  | 0.176 |  |  |
|  | CC + CT | 15 | 3.27 | 3.94 | 36 | 64.96 | 192.56 | **<0.001** | 17 | 73.33 | 239.80 | 19 | 57.47 | 144.42 | 0.168 |
|  | TT | 10 | 2.95 | 1.57 | 67 | 53.56 | 179.92 | **0.005** | 28 | 39.90 | 188.22 | 39 | 63.37 | 175.54 | **<0.001** |
|  | P-value |  | 0.505 |  |  | 0.148 |  |  |  | **0.017** |  |  | 0.842 |  |  |
|  | **Male** |  |  |  |  |  |  |  |  |  |  |  |  |  |  |
|  | C | 5 | 3.27 | 1.83 | 54 | 65.64 | 191.56 | **0.023** | 19 | 5.76 | 8.11 | 35 | 98.14 | 232.54 | **<0.001** |
|  | T | 17 | 6.16 | 4.88 | 131 | 58.85 | 147.97 | **0.010** | 51 | 18.66 | 48.91 | 80 | 84.47 | 181.13 | **<0.001** |
|  | P-value |  | 0.249 |  |  | 0.916 |  |  |  | 0.264 |  |  | 0.722 |  |  |

ACE: Angiotensin-converting enzyme; IL: Interleukin.

Supplementary Table S10: Association ACE1 I/D and ACE2 rs1978124 Genotypes/ Alleles Distribution with Serum IL-6 Levels.

| Genotypes  Alleles | | IL-6 (Ng/L) | | | | | | | | | | | | | |
| --- | --- | --- | --- | --- | --- | --- | --- | --- | --- | --- | --- | --- | --- | --- | --- |
|  |  | Inpatient  ICU-No | | | Inpatient  ICU-Yes | | | P-value | Inpatient  Intubated -No | | | Inpatient  Intubated -Yes | | | P-value |
|  |  | N | Mean | SD | N | Mean | SD |  | N | Mean | SD | N | Mean | SD |  |
| **ACE1 I/D** | II | 30 | 64.15 | 183.52 | 14 | 60.12 | 103.00 | 0.208 | 33 | 59.26 | 175.41 | 11 | 73.69 | 113.31 | 0.076 |
|  | ID | 54 | 39.34 | 77.00 | 28 | 186.93 | 288.16 | **0.002** | 61 | 41.12 | 76.19 | 21 | 230.96 | 319.88 | **0.001** |
|  | DD | 38 | 29.51 | 38.15 | 9 | 278.88 | 419.87 | 0.204 | 42 | 33.95 | 53.71 | 5 | 441.10 | 514.23 | 0.091 |
|  | P-value |  | 0.767 |  |  | 0.444 |  |  |  | 0.531 |  |  | 0.385 |  |  |
|  | II | 30 | 64.15 | 183.52 | 14 | 60.12 | 103.00 | 0.208 | 33 | 59.26 | 175.41 | 11 | 73.69 | 113.31 | 0.076 |
|  | ID + DD | 92 | 35.28 | 63.78 | 37 | 209.29 | 321.02 | **0.001** | 103 | 38.19 | 67.72 | 26 | 271.37 | 362.36 | **<0.001** |
|  | P-value |  | 0.651 |  |  | 0.225 |  |  |  | 0.445 |  |  | 0.212 |  |  |
|  | II + ID | 84 | 48.20 | 125.28 | 42 | 144.66 | 248.41 | **0.001** | 94 | 47.49 | 120.03 | 32 | 176.90 | 275.53 | **<0.001** |
|  | DD | 38 | 29.51 | 38.15 | 9 | 278.88 | 419.87 | 0.204 | 42 | 33.95 | 53.71 | 5 | 441.10 | 514.23 | 0.091 |
|  | P-value |  | 0.723 |  |  | 0.980 |  |  |  | 0.634 |  |  | 0.374 |  |  |
| **ACE2 rs1978124** | **Female** |  |  |  |  |  |  |  |  |  |  |  |  |  |  |
|  | CC | 2 | 14.55 | 1.77 | 1 | 641.00 | . | 0.221 | 2 | 14.55 | 1.77 | 1 | 641.00 | . | 0.221 |
|  | CT | 9 | 21.43 | 31.16 | 7 | 32.71 | 35.37 | 0.266 | 10 | 20.40 | 29.56 | 6 | 36.31 | 37.31 | 0.329 |
|  | TT | 30 | 20.69 | 28.10 | 9 | 205.61 | 337.51 | 0.072 | 33 | 19.35 | 27.11 | 6 | 305.48 | 382.55 | **0.001** |
|  | P-value |  | 0.851 |  |  | 0.284 |  |  |  | 0.560 |  |  | 0.122 |  |  |
|  | CC | 2 | 14.55 | 1.77 | 1 | 641.00 | . | 0.221 | 2 | 14.55 | 1.77 | 1 | 641.00 | . | 0.221 |
|  | TT + CT | 39 | 20.86 | 28.41 | 16 | 129.97 | 262.87 | 0.052 | 43 | 19.59 | 27.34 | 12 | 170.90 | 294.81 | **0.004** |
|  | P-value |  | 0.363 |  |  | 0.153 |  |  |  | 0.295 |  |  | 0.181 |  |  |
|  | CC + CT | 11 | 20.18 | 28.01 | 8 | 108.74 | 217.54 | 0.186 | 12 | 19.42 | 26.84 | 7 | 122.69 | 231.08 | 0.176 |
|  | TT | 30 | 20.69 | 28.10 | 9 | 205.61 | 337.51 | 0.072 | 33 | 19.35 | 27.11 | 6 | 305.48 | 382.55 | **0.001** |
|  | P-value |  | 0.894 |  |  | 0.773 |  |  |  | 0.888 |  |  | 0.253 |  |  |
|  | **Male** |  |  |  |  |  |  |  |  |  |  |  |  |  |  |
|  | C | 25 | 76.91 | 197.58 | 10 | 151.23 | 309.52 | 0.411 | 31 | 73.24 | 182.49 | 4 | 291.15 | 473.04 | 0.066 |
|  | T | 56 | 42.95 | 79.63 | 24 | 181.37 | 289.01 | 0.008 | 60 | 45.80 | 80.16 | 20 | 200.50 | 312.35 | **0.022** |
|  | P-value |  | 0.408 |  |  | 0.533 |  |  |  | 0.569 |  |  | 0.561 |  |  |

ACE: Angiotensin-converting enzyme; IL: Interleukin.

Supplementary Table S11: Association ACE1 I/D and ACE2 rs1978124 Genotypes/ Alleles Distribution with Serum IL-6 Levels.

| Genotypes  Alleles | | IL-6 (Ng/L) | | | | | | |
| --- | --- | --- | --- | --- | --- | --- | --- | --- |
|  |  | Survived | | | Deceased | | | P-value |
|  |  | N | Mean | SD | N | Mean | SD |  |
| **ACE1 I/D** | II | 56 | 54.07 | 187.36 | 13 | 71.22 | 104.91 | **0.002** |
|  | ID | 106 | 49.79 | 150.20 | 21 | 173.18 | 265.60 | **<0.001** |
|  | DD | 85 | 23.31 | 49.59 | 7 | 333.07 | 458.60 | **0.001** |
|  | P-value |  | 0.384 |  |  | 0.383 |  |  |
|  | II | 56 | 54.07 | 187.36 | 13 | 71.22 | 104.91 | **0.002** |
|  | ID + DD | 191 | 38.00 | 117.17 | 28 | 213.15 | 322.43 | **<0.001** |
|  | P-value |  | 0.959 |  |  | 0.245 |  |  |
|  | II + ID | 162 | 51.27 | 163.43 | 34 | 134.20 | 222.00 | **<0.001** |
|  | DD | 85 | 23.31 | 49.59 | 7 | 333.07 | 458.60 | **0.001** |
|  | P-value |  | 0.814 |  |  | 0.051 |  |  |
| **ACE2 rs1978124** | **Female** |  |  |  |  |  |  |  |
|  | CC | 3 | 10.37 | 7.35 | 1 | 641.00 | . | 0.180 |
|  | CT | 28 | 52.02 | 187.36 | 4 | 52.47 | 35.69 | **0.023** |
|  | TT | 60 | 29.11 | 129.28 | 7 | 263.14 | 366.75 | **0.001** |
|  | P-value |  | 0.407 |  |  | 0.393 |  |  |
|  | CC | 3 | 10.37 | 7.35 | 1 | 641.00 | . | 0.180 |
|  | TT + CT | 88 | 36.40 | 149.48 | 11 | 186.54 | 303.94 | **<0.001** |
|  | P-value |  | 0.678 |  |  | 0.192 |  |  |
|  | CC + CT | 31 | 47.99 | 178.19 | 5 | 170.18 | 265.01 | **0.006** |
|  | TT | 60 | 29.11 | 129.28 | 7 | 263.14 | 366.75 | **0.001** |
|  | P-value |  | 0.180 |  |  | 0.935 |  |  |
|  | **Male** |  |  |  |  |  |  |  |
|  | C | 48 | 67.59 | 203.16 | 6 | 50.00 | 26.04 | **0.017** |
|  | T | 108 | 35.26 | 76.39 | 23 | 169.62 | 292.29 | **<0.001** |
|  | P-value |  | 0.855 |  |  | 0.726 |  |  |

ACE: Angiotensin-converting enzyme; IL: Interleukin.

Supplementary Table S12: Association ACE1 I/D and ACE2 rs1978124 Genotypes/ Alleles Distribution with Serum ACE1 Levels.

| Genotypes  Alleles | | ACE1 (U/L) | | | | | | | | | | | | | |
| --- | --- | --- | --- | --- | --- | --- | --- | --- | --- | --- | --- | --- | --- | --- | --- |
|  |  | Control | | | COVID-19 | | | P-value | Outpatient | | | Inpatient | | | P-value |
|  |  | N | Mean | SD | N | Mean | SD |  | N | Mean | SD | N | Mean | SD |  |
| **ACE1 I/D** | II | 9 | 39.22 | 17.91 | 96 | 51.21 | 37.85 | 0.426 | 41 | 39.88 | 13.96 | 55 | 59.65 | 46.97 | **0.008** |
|  | ID | 30 | 50.13 | 15.88 | 192 | 65.21 | 63.79 | 0.665 | 80 | 48.09 | 18.96 | 112 | 77.44 | 79.90 | **0.007** |
|  | DD | 17 | 60.94 | 13.90 | 133 | 66.78 | 54.25 | 0.510 | 61 | 56.15 | 19.41 | 72 | 75.79 | 70.52 | 0.163 |
|  | P-value |  | **0.011** |  |  | **<0.001** |  |  |  | **<0.001** |  |  | **0.038** |  |  |
|  | II | 9 | 39.22 | 17.91 | 96 | 51.21 | 37.85 | 0.426 | 41 | 39.88 | 13.96 | 55 | 59.65 | 46.97 | **0.008** |
|  | ID + DD | 47 | 54.04 | 15.93 | 325 | 65.85 | 59.99 | 0.867 | 141 | 51.57 | 19.51 | 184 | 76.79 | 76.17 | **0.003** |
|  | P-value |  | 0.052 |  |  | <0.001 |  |  |  | <0.001 |  |  | 0.032 |  |  |
|  | II + ID | 39 | 47.62 | 16.78 | 288 | 60.54 | 56.80 | 0.632 | 121 | 45.31 | 17.80 | 167 | 71.58 | 71.11 | **<0.001** |
|  | DD | 17 | 60.94 | 13.90 | 133 | 66.78 | 54.25 | 0.510 | 61 | 56.15 | 19.41 | 72 | 75.79 | 70.52 | 0.163 |
|  | P-value |  | **0.006** |  |  | **<0.001** |  |  |  | **<0.001** |  |  | **0.038** |  |  |

ACE: Angiotensin-converting enzyme.

Supplementary Table S13: Association ACE1 I/D and ACE2 rs1978124 Genotypes/ Alleles Distribution with Serum ACE1 Levels.

| Genotypes  Alleles | | ACE1 (U/L) | | | | | | | | | | | | | |
| --- | --- | --- | --- | --- | --- | --- | --- | --- | --- | --- | --- | --- | --- | --- | --- |
|  |  | Inpatient  ICU-No | | | Inpatient  ICU-Yes | | | P-value | Inpatient  Intubated -No | | | Inpatient  Intubated -Yes | | | P-value |
|  |  | N | Mean | SD | N | Mean | SD |  | N | Mean | SD | N | Mean | SD |  |
| **ACE1 I/D** | II | 38 | 59.89 | 49.32 | 17 | 59.12 | 42.64 | 0.935 | 44 | 58.20 | 46.14 | 11 | 65.45 | 52.07 | 0.697 |
|  | ID | 77 | 72.36 | 59.67 | 35 | 88.60 | 112.68 | 0.321 | 89 | 76.01 | 73.47 | 23 | 82.96 | 102.86 | 0.476 |
|  | DD | 59 | 79.29 | 76.20 | 13 | 59.92 | 31.98 | 0.185 | 67 | 78.19 | 72.47 | 5 | 43.60 | 14.52 | **0.046** |
|  | P-value |  | **0.022** |  |  | 0.919 |  |  |  | **0.010** |  |  | 0.804 |  |  |
|  | II | 38 | 59.89 | 49.32 | 17 | 59.12 | 42.64 | 0.935 | 44 | 58.20 | 46.14 | 11 | 65.45 | 52.07 | 0.697 |
|  | ID + DD | 136 | 75.37 | 67.16 | 48 | 80.83 | 98.04 | 0.099 | 156 | 76.95 | 72.81 | 28 | 75.93 | 94.28 | 0.099 |
|  | P-value |  | **0.013** |  |  | 0.875 |  |  |  | **0.008** |  |  | 0.791 |  |  |
|  | II + ID | 115 | 68.24 | 56.55 | 52 | 78.96 | 96.07 | 0.418 | 133 | 70.12 | 66.05 | 34 | 77.29 | 89.13 | 0.703 |
|  | DD | 59 | 79.29 | 76.20 | 13 | 59.92 | 31.98 | 0.185 | 67 | 78.19 | 72.47 | 5 | 43.60 | 14.52 | **0.046** |
|  | P-value |  | **0.038** |  |  | 0.682 |  |  |  | **0.018** |  |  | 0.515 |  |  |

ACE: Angiotensin-converting enzyme.

Supplementary Table S14: Association ACE1 I/D and ACE2 rs1978124 Genotypes/ Alleles Distribution with Serum ACE1 Levels.

| Genotypes  Alleles | | ACE1 (U/L) | | | | | | |
| --- | --- | --- | --- | --- | --- | --- | --- | --- |
|  |  | Survived | | | Deceased | | | P-value |
|  |  | N | Mean | SD | N | Mean | SD |  |
| **ACE1 I/D** | II | 83 | 48.70 | 35.04 | 13 | 67.23 | 51.29 | 0.252 |
|  | ID | 165 | 61.45 | 55.63 | 27 | 88.15 | 98.66 | 0.382 |
|  | DD | 126 | 67.44 | 55.33 | 7 | 54.86 | 27.83 | 0.397 |
|  | P-value |  | **<0.001** |  |  | 0.896 |  |  |
|  | II | 83 | 48.70 | 35.04 | 13 | 67.23 | 51.29 | 0.252 |
|  | ID + DD | 291 | 64.05 | 55.49 | 34 | 81.29 | 89.42 | 0.895 |
|  | P-value |  | **<0.001** |  |  | 0.877 |  |  |
|  | II + ID | 248 | 57.19 | 49.99 | 40 | 81.35 | 86.01 | 0.150 |
|  | DD | 126 | 67.44 | 55.33 | 7 | 54.86 | 27.83 | 0.397 |
|  | P-value |  | **<0.001** |  |  | 0.698 |  |  |

ACE: Angiotensin-converting enzyme.
